# TLR4-Mediated Blunting of Inflammatory Responses to Eccentric Exercise in Young Women

**DOI:** 10.1155/2014/479395

**Published:** 2014-09-09

**Authors:** Rodrigo Fernandez-Gonzalo, José A. De Paz, Paula Rodriguez-Miguelez, María J. Cuevas, Javier González-Gallego

**Affiliations:** ^1^Institute of Biomedicine (IBIOMED), University of León, Campus Universitario, 24071 León, Spain; ^2^Department of Physiology and Pharmacology, Karolinska Institutet, Stockholm 171 77, Sweden

## Abstract

This study assessed the inflammatory response mediated by the toll-like receptor 4 (TLR4) signaling pathway after acute eccentric exercise before and after an eccentric training program in women. Twenty women performed two acute eccentric bouts using a squat machine over a ~9 week interval. The training group (TG) carried out an eccentric training program during 6 weeks, while the control group (CG) did not follow any training. Protein content of markers involved in the TLR4-mediated activation of several nuclear transcription factors, such as nuclear factor *κ*B (NF-*κ*B), and interferon regulatory transcription factor 3 (IRF3), was analyzed. The inflammatory response after the first acute bout was similar between TG and CG, showing an upregulation of all the markers analyzed, with the exception of IRF3. After the second bout, the upregulation of TLR4 signaling pathway was blunted in TG, but not in CG, through both the myeloid differentiation factor 88- and toll/interleukin-1 receptor domain containing adapter inducing interferon-*β*-dependent pathways. These results highlight the role of the TLR4 in controlling the exercise-induced inflammatory response in young women. More importantly, these data suggest eccentric training may help to prevent TLR4 activation principally through NF-*κ*B, and perhaps IRF3, downstream signaling in this population.

## 1. Introduction

Regular exercise offers protection against and may be useful as a treatment for a wide variety of chronic diseases associated with low-grade inflammation [1–3]. Indeed, exercise triggers important adaptations in the inflammatory system [4], which vary depending on the type and duration of the exercise intervention. Although the inflammatory response to acute exercise may not fully resemble disease associated inflammation, it represents a well-established and useful model to investigate the mechanisms controlling inflammation. In particular, studies employing eccentric exercise have highlighted the impact of this type of exercise on the inflammatory response [5]. Thus, an acute bout of eccentric exercise induces a marked proinflammatory response, while an eccentric training program attenuates the activation of different signaling pathways involved in inflammatory processes [6–8].

Recently, the regulation of toll-like receptors (TLRs) as key factors involved in the inflammatory responses to exercise has received great attention [9, 10]. TLRs are type-I transmembrane glycoproteins expressed in different cells of the innate immune system and in other cell types, such as fibroblasts or epithelial cells [11]. TLRs mediate the recognition of pathogen-associated molecular patterns and play an important role in the immune and inflammatory responses [10]. Among all the TLRs described, TLR4 has been shown to highly respond to different exercise protocols [6, 12–14]. In conjunction with CD14, TLR4 orchestrates several processes in the inflammatory cascade [11] and can activate different signaling pathways that increase, or inhibit, the activity of several transcription factors. Three major transcription factors are controlled, at least to some extent, by TLR4 signaling pathways: nuclear transcription factor *κ*B (NF-*κ*B), activator protein-1 (AP-1), and interferon regulatory transcription factor 3 (IRF3) [15]. A recent study showed that eccentric exercise plays an important role in TLR4-induced NF-*κ*B activation through both the myeloid differentiation factor 88- (MyD88-) dependent and independent pathways in peripheral blood mononuclear cells (PBMC) of young men [6]. However, data explaining possible exercise-induced TLR4 regulation of AP-1 and IRF3 are scarce.

Most of the TLR4 signaling data have been collected with elderly populations [10, 16–18], and although few reports include young men [6, 19], TLR4 response to exercise is practically unknown in young women. This may seem surprising given that women are more susceptible to autoimmune and inflammatory diseases, probably due to their higher basal inflammatory status [20, 21]. In fact, the sex difference in subcutaneous adiposity and a differential regulation of cytokine production are probably important factors explaining the sex differences in proinflammatory markers concentrations [22, 23]. Given the great benefits of exercise as a potential tool to protect against the development of chronic inflammatory diseases later in life [24], research assessing exercise-induced TLR4 activity in young women is warranted.

Therefore, this study was designed to complete two purposes. First, we aimed to describe the regulation of NF-*κ*B, IRF3, and indirectly AP-1 through TLR4 signaling pathway activation in PBMC in response to an acute bout of eccentric exercise in young female subjects. Considering that TLR4 signaling was upregulated in men after a similar bout of exercise [6], we hypothesized that the acute eccentric bout would induce an upregulation of the TLR4-related pathways. In addition, to further understand the effects of eccentric training on TLR4 regulation, we evaluated the influence of this training paradigm on the activity pattern of different TLR4-initiated pathways. It was hypothesized that the activation of the different TLR4 signaling pathways, for example, NF-*κ*B, AP-1, and IRF3, in response to an acute eccentric bout would be reduced after the eccentric training period.

## 2. Materials and Methods

### 2.1. Study Design

All the activities involved in this study, including familiarization sessions, were performed during a 12-week period. Initially, two groups of young women completed maximal strength tests, followed by an acute eccentric bout some days later. After 2 weeks, one of the groups (training group, TG) followed a 6-week eccentric training program, while the rest of the participants (control group, CG) followed their daily routines. One week after the last training session, all the subjects repeated the strength tests and the acute eccentric bout. Blood samples were obtained before and immediately after and 2 h after each acute eccentric bout. Peripheral blood mononuclear cells (PBMC) were isolated to subsequently analyze the TLR4 signaling pathway responses.

### 2.2. Subjects

Twenty young active women completed the study. All subjects were healthy sport science undergraduate students involved in recreational physical activities 3–5 h per week (team and ball sports). None of them reported previous or present resistance exercise training experience or muscle joint or bone injuries. Subjects reporting inflammatory or autoimmune conditions or taking any medication known to affect their inflammatory, hormonal, or metabolic response were excluded from the study. None of the participants were smokers. To minimize the effects of different sex hormone concentrations, for example, estrogens may modulate innate immunity both at rest and during exercise [20, 23], all the volunteers from the present investigation carried out the first and the second (±1 week) acute eccentric bouts during the follicular phase of the menstrual cycle (self-reported). This phase has been described as an appropriate moment to evaluate the effects of exercise in women [23, 25]. Participants were randomly assigned to either a training group (TG; *n* = 12) or to a control group (CG; *n* = 8). Age, height, body mass, body mass index, and fat % (analyzed by skinfold measurements using Yuhasz equation) were 22.5 ± 0.3 yrs, 163.8 ± 1.4 cm, 60 ± 2.1 kg, 22.4 ± 3.2 kg/m^2^, and 18.5 ± 2.8% fat, respectively, in women from TG and 22.5 ± 2.3 yrs, 160.7 ± 1.8 cm, 59.7 ± 3.1 kg, 23.0 ± 2.5 kg/m^2^, and 17.9 ± 3.1% fat, respectively, in women form CG. The purposes and possible risks associated with participation in the study were explained to the subjects before written consent for participation was obtained. The study followed the principles of the Declaration of Helsinki, and all procedures were approved by the local ethics committee.

### 2.3. Maximal Strength Assessment

Maximal strength tests took place after two familiarization sessions, where appropriate squat exercise technique was explained. Three to five days before the first and the second acute eccentric bouts all participants performed a maximal voluntary isometric contraction (MVIC) and a one repetition maximum (1RM) tests using a multipower device (e.g., guided barbell squat exercise, Salter, Barcelona, Spain). After a standardized warmup, subjects carried out the MVIC test in a squat position, with 110° knee flexion. Each participant completed two MVIC tests lasting 5 seconds, with 1 min of rest in between. Force was registered using a strain gauge (Globus Ergometer, Codognè, Italy). The greatest force value obtained was considered as the MVIC. If the values obtained after the two trials differed more than 5%, a third test was performed. After ~30 min of rest, 1RM squat test was completed. Briefly, participants had to lift an estimated load from 90° knee flexion to full extension (180°). The load was increased 10 kg if the participant succeeded or decreased 5 kg if they failed. All participants achieved their 1RM in 3 to 5 attempts. A recovery period of 3 min was allowed between two attempts.

### 2.4. Acute Eccentric Bouts

The protocol followed during the acute eccentric-damaging bouts was similar to Fernández-Gonzalo et al. and García-López et al. [6, 7]. The eccentric insult corresponded to the negative phase of the squat exercise performed using a guided barbell multipower device. The bout comprised 12 sets of 10 repetitions with a load equivalent to 60% of the MVIC. A 3-min rest period was allowed between sets. Once the subject completed the eccentric action (lower the load from 180°, full extension, to 90° knee flexion), research assistants raised the load up using a pulley system [26]. Subjects lowered the load with the most comfortable velocity for them, although they were requested to control the descent of the barbell during the full range of motion, allowing the subjects to stop the movement at ~90° knee flexion. An encoder system (Globus Real Power) was used to register distance, time, and velocity of the vertical displacements of the barbell. The first acute eccentric bout was performed ~2 weeks before the first training session, whereas the second bout was carried out ~1 week after the last training session. Muscle soreness was assessed using a visual analogue scale. All participants completed both acute eccentric bouts.

### 2.5. Eccentric Training 

Over 6 weeks, TG subjects completed 18 training sessions (3 sessions per week) with at least 48 h between sessions. The training program was initiated ~2 weeks after the first acute eccentric bout and it was completed ~1 week before the second acute eccentric bout. The eccentric action (full extension to 90° knee flexion) and the multipower device described for the acute eccentric bouts were used during the training sessions. The velocity of each eccentric action was monitored to offer real-time feedback to the subjects, allowing them to perform the movement with a similar velocity to the initial acute bout. The load and volume of the training was progressively increased in a weekly manner: weeks 1 and 2: 3 × 10 and 5 × 10 at 40% of MVIC, respectively; weeks 3 and 4: 3 × 10 and 5 × 10 at 45% of MVIC, respectively; weeks 5 and 6: 3 × 10 and 5 × 10 at 50% of MVIC, respectively.

### 2.6. Blood Sample Preparation

Blood samples (30 mL) were obtained from the brachiocephalic vein before, immediately after, and 2 h after each acute eccentric bout using Vacutainer system (BD, Franklin Lakes, NJ) with EDTA. Basal blood collection was performed under conditions of fasting and rest (8:00 a.m). All participants remained in the Exercise Physiology Laboratory at the University of León until finishing acute testing and sampling. During this time, the subjects ingested only water. No caffeine or alcohol was allowed 48 hrs before the acute eccentric bouts. Relative quantity of PBMC subpopulations was assessed using an automatic analyzer in 5 mL of total blood (Technicon H1, Bayer, Tarrytwon, NY). Density gradient centrifugation on Ficoll separating solution (Biochrom AG, Berlin, Germany) was employed to separate PBMC from total blood [27].

### 2.7. Reverse Transcription and Quantitative Real-Time Polymerase Chain Reaction

Total RNA was isolated from PBMC using a RiboPureTM-Blood Kit (Ambion, Paisley, UK) and quantified by spectrophotometry (NanoDrop 1000, Thermo Scientific, Waltham, MA, USA). DNase I (RNase-free) (Ambion) was used to removed residual genomic DNA. First-standard cDNA was synthesized using High-Capacity cDNA Archive Kit (Applied Biosystems, Paisley, UK) and then it was amplified using TaqMan Universal PCR Master Mix (Applied Biosystems) on a StepOnePlusTM Real-Time PCR Systems (Applied Biosystems). TaqMan primers and probes for CD14 (Genbank M86511.1 and Hs00169122_g1), TLR4 (Genbank U88880.1 and Hs00370853_m1), TRAF6 (Genbank BC031052.1 and Hs00377558_m1), and 18SrRNA as housekeeping gene (Genbank X03205.1 and Hs99999901_s1) were derived from the commercially available TaqMan Assays-on-Demand Gene (Applied Biosystems). Relative changes in gene expression levels were determined using the 2-ΔΔCT method as described previously [6]. The cycle number at which the transcripts were detectable (CT) was normalized to the cycle number of GAPDH detection, referred to as ΔCT.

### 2.8. Western Blot Analysis

Western blot analyses were performed in PBMC or nuclear extracts homogenates as described previously [28]. Lysate proteins were fractionated by SDS-PAGE and Western blotting was performed using the corresponding primary antibodies. Antibodies against TLR4 (96 kDa), MyD88 (33 kDa), p65 (65 kDa), pERK (42–44 kDa), TBK1 (80 kDa), IRF3 (50 kDa), protein C-reactive (PCR) (30 kDa), and TNF*α* (27 kDa) were purchased from Santa Cruz Biotechnology (Santa Cruz, CA); antibodies against CD14 (40 kDa), TRAF6 (55 kDa), TRIF (66 kDa), and IKKi/IKK*ε* (80 kDa) were purchased from Abcam (Cambridge, UK); and antibodies against pIKK (85–87 kDa), pI*κ*B (40 kDa), p38 (43 kDa), pP38 (43 kDa), and pIRF3 (45–55 kDa) were purchased from Cell Signaling Technology (Beverly, MA). Bound antibody was detected by enhanced chemiluminescence.

Equal loading of protein was demonstrated by probing the membranes with a rabbit antilamin-B polyclonal antibody (Santa Cruz Biotechnology) (67 kDa) or rabbit anti-*β*-actin polyclonal antibody (Sigma-Aldrich) (42 kDa). The density of the specific bands was quantified with an imaging densitometer (Image J, National Institute of Health, Bethesda, MD).

### 2.9. Statistical Analysis

Data are expressed as means of percentages from resting values ± standard error of means (SEM). Shapiro-Wilk test was used to verify normal data distribution. To compare differences within each group, data were analyzed using an analysis of variance (ANOVA) with repeated measures for training (pre- and posttraining) and time (baseline, immediately, and 2 h after acute bout). To compare results between TG and CG after the first and the second acute eccentric bout, a two-way ANOVA with repeated measurements for group (TG and CG) and time (baseline, immediately, and 2 h after acute bouts) was used. Bonferroni* post hoc* analysis was used where appropriate. A value of *P* < 0.05 was regarded as significant. All analyses were performed with SPSS version 17.0 statistical software (Chicago, IL).

## 3. Results

### 3.1. Effects of Eccentric Training on Functional Parameters and Leukocyte Subpopulations Distribution

The 6-week eccentric training program took place during the 9-week interval between the two acute eccentric bouts. At baseline, there were no differences in 1RM or MVIC between groups (*P* > 0.05). One repetition maximum (126 ± 4 versus 140 ± 5 Kg) and MVIC (140 ± 4 versus 159 ± 3 Kg) increased in TG after the training program, but not in CG (137 ± 7 versus 139 ± 7 Kg and 149 ± 8 versus 142 ± 7 Kg, for 1RM and MVIC, resp). No differences between exercise bouts or groups were found in the distribution of PBMC subpopulations (Table 1). Muscle soreness (mm in VAS) before and 48 hours after the first acute eccentric bout was 2.9 ± 5.3 and 43.6 ± 16.9, respectively, for TG and 2.5 ± 4.3 and 49.3 ± 16.3, respectively, for CG. Compared with the first, soreness was lower in both groups (*P* < 0.05) 48 hours after the second acute eccentric bout (13.0 ± 5.7 for TG; 31.7 ± 9.1 for CG), and the decrease was greater for the TG (*P* < 0.001).

### 3.2. Effects of Eccentric Training on TLR4 and CD14 Expression

**Figure 1:**
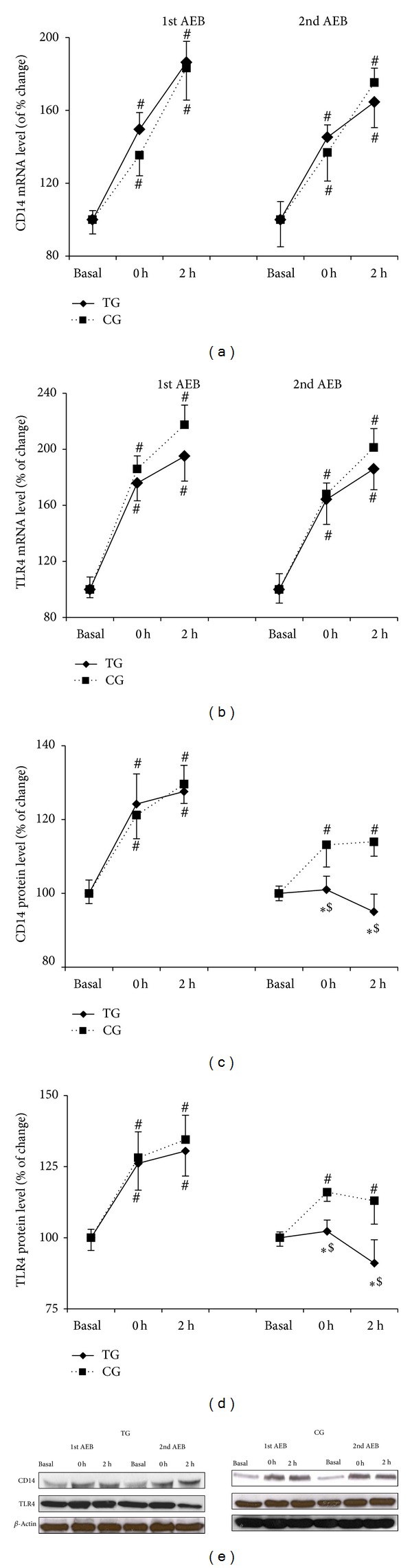
Training reduces CD14 and TLR4 protein overexpression induced by acute eccentric exercise. CD14 (a) and TLR4 (b) mRNA levels, CD14 (c) and TLR4 (d) protein contents after the first and the second acute eccentric bout (AEB) for training group (TG, solid line) and control group (CG, dotted line), and representative Western blots (e). Mean ± SEM. ∗*P* < 0.05 versus CG; ^#^ *P* < 0.05 versus basal value, same group; ^$^ *P* < 0.05 versus 1st acute eccentric bout, same group.

CD14 and TLR4 mRNA levels increased after both acute eccentric bouts for TG and CG (*P* < 0.04), with no differences between bouts or groups (Figures 1(a) and 1(b)). The first acute bout of eccentric exercise induced an increase of CD14 and TLR4 protein content in CG and TG that was significant immediately after exercise (*P* < 0.05) and after 2 h (*P* < 0.05). Similar results were observed after the second bout in CG group. However, after the training program no significant change was detected following the eccentric bout in TG (Figures 1(c) and 1(d)).

### 3.3. Effects of Eccentric Training on MyD88-Dependent Pathway

**Figure 2:**
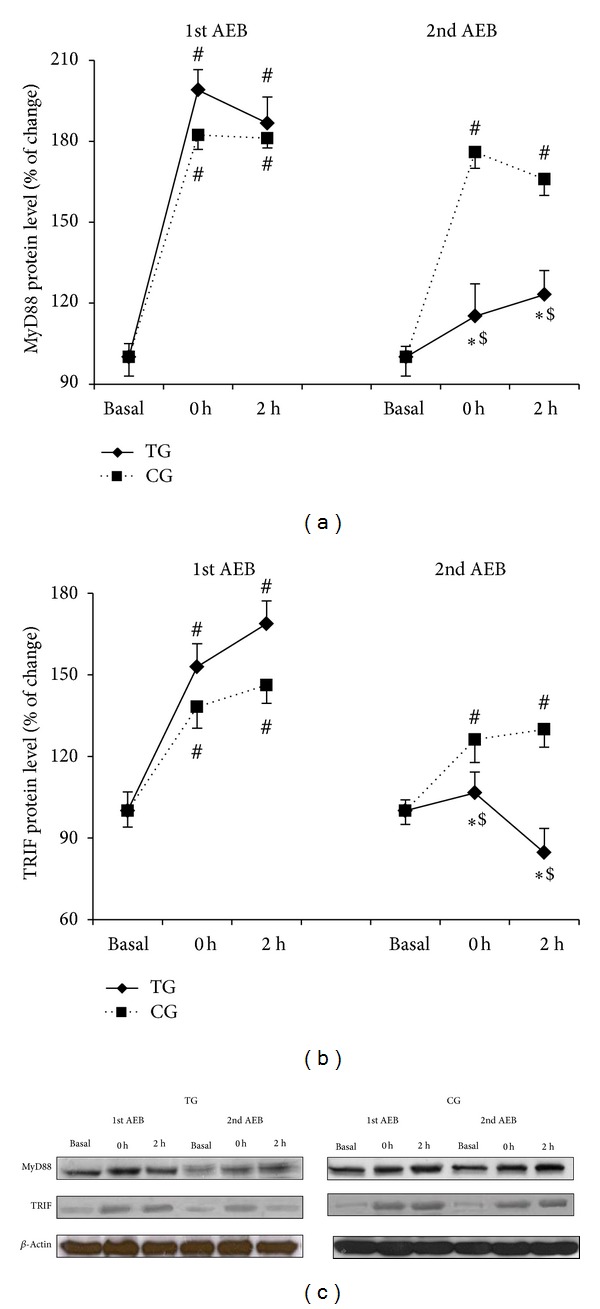
Training reduces MyD88 and TRIF protein overexpression induced by acute eccentric exercise. MyD88 (a) and TRIF (b) protein levels after the first and the second acute eccentric bout (AEB) for training group (TG, solid line) and control group (CG, dotted line) and representative Western blots (c). Mean ± SEM. ∗*P* < 0.05 versus CG; ^#^ *P* < 0.05 versus basal value, same group; ^$^ *P* < 0.05 versus 1st acute eccentric bout, same group.

MyD88 (Figure 2(a)) increased after the first acute bout in TG and CG (*P* < 0.02). However, this protein was downregulated after the second bout in TG when compared with the first eccentric bout and with CG (*P* < 0.03). Similarly, TRIF (Figure 2(b)) also increased significantly (*P* < 0.03) in response to the first acute eccentric bout in both CG and TG, but only in CG after the second bout. TRIF protein expression was significantly reduced after the second bout in TG (*P* < 0.01) when compared with the first bout or with CG.

**Figure 3:**
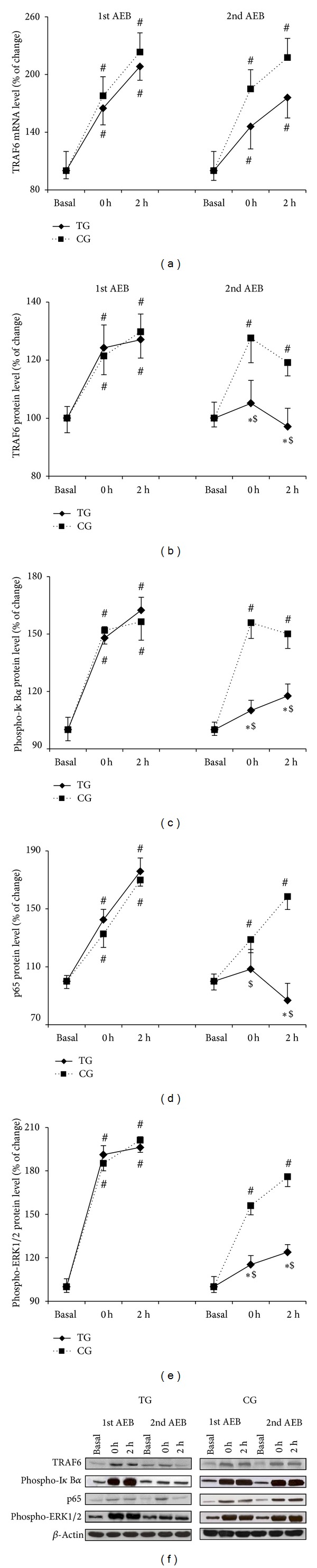
Training reduces TRAF6 and p65 protein overexpression and I*κ*B*α* and ERK1/2 phosphorylation after eccentric exercise. TRAF6 mRNA (a), TRAF6 protein (b), phospho-I*κ*B*α* (c), p65 (d), and phospho-ERK1/2 (e) protein levels after the first and the second acute eccentric bout (AEB) for training group (TG, solid line) and control group (CG, dotted line) and representative Western blots (f). Mean ± SEM. ∗*P* < 0.05 versus CG; ^#^ *P* < 0.05 versus basal value, same group; ^$^ *P* < 0.05 versus 1st acute eccentric bout, same group.

TRAF6 mRNA levels were also analyzed. In the same line with CD14 and TLR4, TRAF6 mRNA levels (Figure 3(a)) increased after both acute eccentric bouts for TG and CG (*P* < 0.05). The first acute eccentric bout triggered a significant (*P* < 0.04 immediately after and *P* < 0.03 2 h after the first acute eccentric bout) increase in TRAF6 protein concentration (Figure 3(b)) in TG and CG. After the second acute bout, CG still showed such upregulation, while TG values did not differ from basal. Phospho-I*κ*B*α* protein levels (Figure 3(c)) increased significantly (*P* < 0.02) in response to the first acute eccentric bout in both CG and TG. Similar results were observed after the second bout in CG group, but a reduction in phospho-I*κ*B*α* was found after the second bout in TG (*P* < 0.01) when compared with the first bout or with CG.  Figure 3(d) shows p65 protein content in nuclear extracts, which increased progressively reaching maximal levels 2 h after the first acute bout (*P* < 0.01). Posttraining values were lower in TG group, but they remained elevated in CG after the second acute bout. A marked increase of cytosolic phospho-ERK-1/2 protein concentration was evident immediately after the first acute eccentric exercise and was maintained 2 h after (*P* < 0.01 and *P* < 0.01, resp.) in CG and TG group (Figure 3(e)). However, 6 weeks of eccentric training reduced the phosphorylation of ERK-1/2 to basal values after the acute bout in TG.

**Figure 4:**
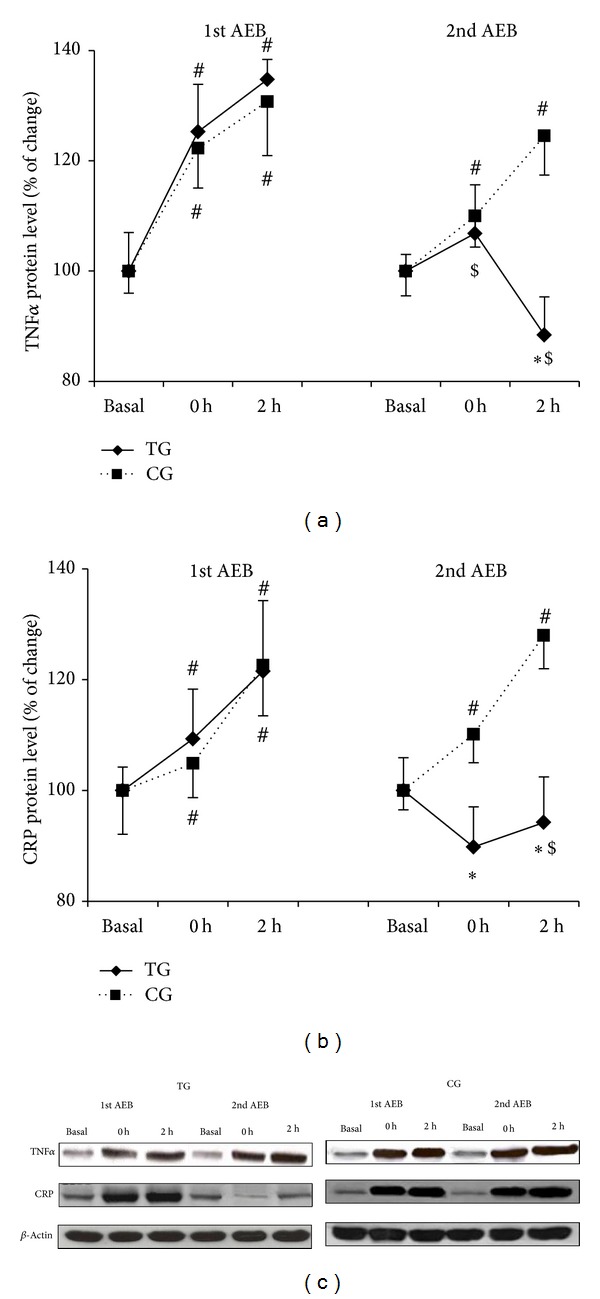
Training reduces TNF*α* and CRP overexpression after eccentric exercise. TNF*α* (a) and CRP (b) protein levels after the first and the second acute eccentric bout (AEB) for training group (TG, solid line) and control group (CG, dotted line), and representative Western blots (c). Mean ± SEM. ∗*P* < 0.05 versus CG; ^#^ *P* < 0.05 versus basal value, same group; ^$^ *P* < 0.05 versus 1st acute eccentric bout, same group.

TNF*α* and CRP protein levels were measured as an estimation of the proinflammatory status (Figures 4(a) and 4(b), resp). TNF*α* increased after the first acute eccentric bout in both TG and CG (*P* < 0.04), but only in CG after the second bout (*P* < 0.04). Furthermore, TG showed lower TNF*α* protein expression after the second acute eccentric bout when compared with the first bout immediately after and 2 h after, and with CG 2 h after the bout (*P* < 0.05). The first acute eccentric bout also triggered a significant increase of CRP (*P* < 0.04) in TG and CG. After the second acute bout, CG still showed upregulation of CRP (*P* < 0.04), while TG values were markedly reduced after the second bout (*P* < 0.05).

### 3.4. Effects of Eccentric Training on TRIF-Dependent Pathway

**Figure 5:**
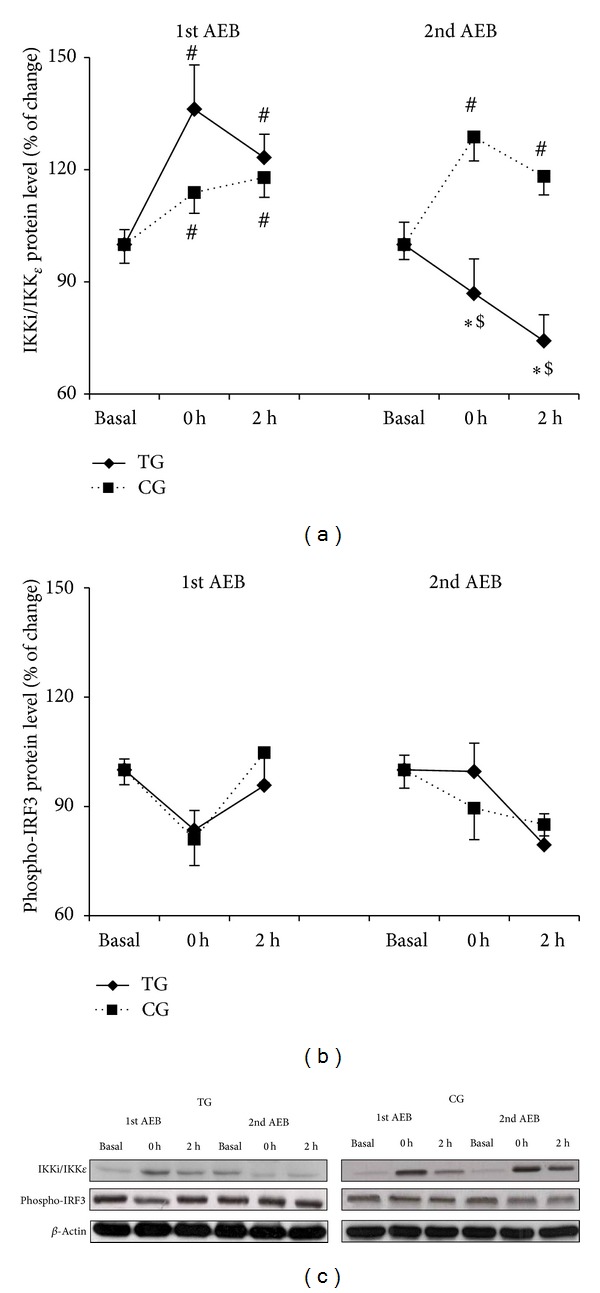
Training attenuates IKKi/IKK*ε* activation induced by acute eccentric exercise. IRF3 phosphorylation is not altered as result of acute eccentric exercise nor training. Total IKKi/IKK*ε* (a) and phospho-IRF3 (b) protein levels after the first and the second acute eccentric bout (AEB) for training group (TG, solid line) and control group (CG, dotted line) and representative Western blots (c). Mean ± SEM. ∗*P* < 0.05 versus CG; ^#^ *P* < 0.05 versus basal value, same group; ^$^ *P* < 0.05 versus 1st acute eccentric bout, same group.

The inducible IKK (Figure 5(a)) increased immediately after the first acute eccentric bout and was maintained 2 h after in both groups (*P* < 0.04). After the second bout this upregulation persisted in CG but was blunted in TG (*P* < 0.03). No changes were observed in the phosphorylation state of IRF3 in response to acute exercise either before or after 6 weeks of training (Figure 5(b)).

## 4. Discussion

The current study investigated the effects of eccentric exercise on the TLR4-mediated inflammatory response in healthy young women. The results indicate that 6 weeks of submaximal eccentric training attenuate several intermediates related to the NF-*κ*B and AP-1 activation and, consequently, the proinflammatory response detected after an acute bout of eccentric exercise. This response was associated with a downregulation of the TLR4 signaling through both MyD88-dependent and independent pathways. Our data further suggest that perhaps other TLR4 signaling pathways, such as IRFs, could be implicated in the beneficial adaptations induced by training.

Previous studies have shown that TLR4 may play a critical role as a link between inflammatory cytokine production and a physically active lifestyle [6, 9, 17]. Thus, resistance-trained older women had significantly lower inflammation and TLR4 mRNA levels than sedentary counterparts. Moreover, physical activity status, but not age, influenced TLR4 cell-surface expression [17]. Our results confirm that a single bout of eccentric exercise increases TLR4 and CD14 mRNA and protein contents in young women, as it had previously been reported in men [6]. Interestingly, only the protein content overexpression of the receptor and coreceptor in PBMC was lower following the eccentric training program. Therefore, these data provide further support for a training-induced downregulation of TLR4 and its coreceptor CD14 at protein level. Confirming previous results in young men, there was a discrepancy between CD14 and TLR4 mRNA levels and protein concentration, which may indicate posttranscriptional adaptations of CD14 and TLR4 in response to eccentric training [6].

The complex TLR4 downstream signaling is far from being completely understood. Different stimuli induce TLR4 activation via recruitment of different adaptor/signaling molecules to the cytoplasmic domain of the receptor, thus resulting in different cellular responses [28]. In addition, the cell surface or endosomal localization of TLR4 drives recruitment of MyD88 or toll/interleukin-1 receptor (TIR) domain containing adapter inducing interferon-*β* (TRIF), respectively [29]. In the present study, an acute eccentric bout activated both the MyD88-dependent pathway and the TRIF-dependent pathway, which mediate the early- and late-phase activation of NF-*κ*B, respectively [30]. This activation was blunted in young women after 6 weeks of training, which resembles previous reports in both human and animal models [6, 31].

Early- and late-phase activation of NF-*κ*B through TLR4 signaling is mediated by TNF receptor-associated factor 6 (TRAF6) [32]. TRAF6 recruits the protein kinase complex TAK1 (transforming growth factor beta-activated kinase-1) and TABs (TAK1 binding proteins) [33, 34], which then activate two distinct pathways involving the I*κ*B kinases (IKK) complex (NF-*κ*B activation) and the mitogen-activated protein kinase (MAPK) (ERK, JNK, p38) pathway, for example, AP-1 activation. The results from the present study indicate that an acute bout of eccentric exercise induced an increase of TRAF6 protein concentration, which was associated with IKK and MAPK signaling pathway activation. This resembles previous observations where eccentric contractions also induced a significant increase in NF-*κ*B activation [7, 8] and MAPKerk1/2 phosphorylation [35]. Remarkably, the eccentric training reduced the protein concentration of TRAF6, phospho-I*κ*B, p65, and MAPKerk1/2 in TG after the second acute eccentric bout compared with the first bout and with CG. The proinflammatory cytokine TNF-*α*, which is regulated by transcription factors such as NF-*κ*B and AP-1 [36], increased its content in PBMC after the first acute bout in both groups. However, this upregulation was blunted by the training program after the second acute bout. CRP data also confirm the anti-inflammatory effects of the training intervention. Taken together, these findings support the role of the eccentric training as a valuable asset to decrease the inflammation associated with acute exercise.

TLR signaling is a tightly regulated process that is controlled at multiple levels. Thus, upon stimulation, TLR4 is internalized into endosomes where two transcriptional events occur: (1) delayed activation of NF-*κ*B and (2) initiation of the IRF3 transcription program. In the latter case, TRIF activates two IKK related proteins through TRAF3, inducible IKK (IKKi or IKK*ε*), and TANK-binding kinase1 (TBK1). IKK*ε* and TBK1 activate interferon regulatory factors, such as IRF3, which dimerise, translocate to the nucleus, and bind interferon response elements (ISRE), resulting in the induction of type-1 interferons [30, 33]. In the present study TRIF protein levels increased after the acute eccentric exercise in parallel to an activation of IKK*ε*, and both effects were inhibited following 6 weeks of submaximal eccentric training. However, our data indicate a nonsignificant increase immediately after and at 2 h after the acute bouts in the IRF3 protein. This discrepancy between IKK*ε* and IRF3 protein concentrations could be due to several reasons. First, we cannot exclude the existence of a methodological limitation since data in the literature show that other authors used a phospho-specific antibody against Ser386 to demonstrate that overexpressed IRF3 is phosphorylated on Ser386 and that phosphorylation on Ser386 is observed in the dimeric form of IRF3 [37, 38]. However, phosphorylation on Ser396 (antibody used in this study) alleviates autoinhibition to allow interaction with CBP (CREB-binding protein) and facilitates phosphorylation at Ser386 [39]. Further studies analyzing IRF3 dimerization are warranted. Second, TBK1 and IKKi can also phosphorylate and activate IRF7, which is the member of IRF family most closely related to IRF3 [40]. Whereas IRF3 is ubiquitously expressed and not inducible, IRF7 is expressed at low levels in most types of cells but is strongly induced in response to various stimuli [41]. However, studies showing the effects of training on IRF7 are still limited. It has been shown that the expression of some downstream effectors of the TLR signaling pathways, such as IRF7, was reduced by addition of resveratrol to exercise training in rat heart [42]. Furthermore, IRF7, which is mainly activated by TLR7 and 9, seems to be the master regulator of type-I interferon-dependent immune responses and exerts transcriptional control over a large set of proinflammatory genes [43]. Thus, IRF7 could play an important role in adaptations induced by training.

The activity profile of the TLR4 pathway may be affected by exercise-induced changes in the PBMC subpopulations. In this study, there were no differences in the distribution of PBMC subpopulations between the first and the second acute eccentric bouts within a group or between groups. Therefore, differences in TLR4 signaling activity between TG and CG and between acute eccentric bouts can only be explained by the eccentric-based training intervention followed by TG. Additionally, it should be noted that none of the proinflammatory markers analyzed were downregulated in the CG. Thus, although the adaptations induced by a single bout of eccentric exercise may decrease the muscle damage from a subsequent bout (i.e., repeated bout effect [44]), it appears that such adaptations do not affect the inflammatory response associated with eccentric exercise in young women. Indeed, the repeated bout effect failed to protect against the proinflammatory response in young men [6, 7].

In summary, this study provides evidences indicating that an acute eccentric exercise increases the TLR4-mediated proinflammatory response through both MyD88- and TRIF-dependent pathways in young women, supporting previous reports in men. More importantly, we show that regular eccentric training may be an effective method to prevent the upregulation of some of the signaling pathways controlling the proinflammatory response in young women. This may be of great importance given the higher risks of women to suffer from autoimmune and inflammatory diseases. Further research assessing long-term effects of eccentric exercise on inflammatory profile in young women is therefore warranted. The present study also supports the notion that TLR4 signaling pathway is a key regulator of the exercise-induced inflammatory response, highlighting the role of TLR4 as a target for future exercise-based anti-inflammatory interventions. However, more research is needed to fully evaluate the potential associations between exercise, TLR4, and activation of the IRF transcription factors.

## 5. Conclusions

The current study provides novel understanding of the molecular mechanisms behind the inflammatory response controlled by the TLR4 signaling pathway after acute exercise in trained and untrained young women. It is suggested that different signaling pathways involving IKK*ε* and the transcription factors IRF are modulated by TLR4 in response to exercise. Additionally, the eccentric training is purported to be a valuable tool to decrease exercise-induced inflammation through a downregulation of the TLR4 downstream signaling in young women. In contrast, the repeated bout effect does not seem to modify the eccentric exercise-induced inflammatory response in women. These data may have implications in the prevention and rehabilitation programs currently employed for autoimmune and inflammatory diseases in this population.

## Figures and Tables

**Table 1:** Distribution of PBMC before (basal) and immediately after (0 h) and 2 hours after (2 h) the acute eccentric bout (AEB) performed before (1st AEB) and after (2nd AEB) the intervention for training group (TG) and control group (CG).

		1st AEB			2nd AEB		
		Basal	0 h	2 h	Basal	0 h	2 h
% Neutrophils	TG	58.8 ± 2.3	63.9 ± 2.3^#^	64.2 ± 0.1^#^	58.0 ± 2.1	62.2 ± 1.6^#^	61.9 ± 1.5^#^
	CG	57.9 ± 2.9	63.8 ± 2.2^#^	63.8 ± 2.1	56.3 ± 4.0	59.9 ± 2.4	61.8 ± 1.0

% Lymphocytes	TG	32.2 ± 2.1	26.7 ± 1.9^#^	27.2 ± 0.8^#^	31.5 ± 2.1	27.3 ± 1.8^#^	29.0 ± 1.5
	CG	32.7 ± 2.2	26.2 ± 1.6^#^	26.6 ± 2.1	32.8 ± 2.9	29.2 ± 1.9	28.7 ± 0.7

% Monocytes	TG	7.2 ± 0.8	7.6 ± 0.9^#^	6.7 ± 0.6	7.5 ± 0.6	7.8 ± 0.5	6.6 ± 0.4^#∗^
	CG	7.1 ± 0.5	7.5 ± 0.5	7.1 ± 0.4	7.9 ± 0.5	7.9 ± 0.6	7.0 ± 0.3

% Eosinophils	TG	1.4 ± 0.3	1.4 ± 0.3	1.4 ± 0.4	2.3 ± 0.7	2.1 ± 0.8	1.9 ± 0.7^#^
	CG	1.8 ± 0.9	1.9 ± 0.8	1.7 ± 0.4	2.4 ± 0.8	2.3 ± 0.5	1.8 ± 0.4

% Basophils	TG	0.4 ± 0.0	0.4 ± 0.0	0.5 ± 0.1	0.7 ± 0.2	0.6 ± 0.1	0.6 ± 0.1
	CG	0.5 ± 0.0	0.6 ± 0.2	0.8 ± 0.1^#^	0.6 ± 0.1	0.7 ± 0.1	0.7 ± 0.2

Mean ± SEM. ^#^ *P* < 0.05 versus basal value; ∗*P* < 0.05 versus 0 h.
